# 3D Analysis of the Cranial and Facial Shape in Craniosynostosis Patients: A Systematic Review

**DOI:** 10.1097/SCS.0000000000010071

**Published:** 2024-03-18

**Authors:** Melissa S.I.C. Kurniawan, Pauline A.E. Tio, Tareq Abdel Alim, Gennady Roshchupkin, Clemens M.F. Dirven, Mieke M. Pleumeekers, Irene M.J. Mathijssen, Marie-Lise C. van Veelen

**Affiliations:** * Department of Plastic and Reconstructive Surgery; † Department of Neurosurgery; ‡ Department of Radiology and Nuclear Medicine, Erasmus University Medical Center; § Department of Epidemiology, Erasmus MC, University Medical Center; ∥ Child Brain Center, Erasmus MC Sophia Children’s Hospital, Rotterdam, The Netherlands

**Keywords:** Cephalometry, craniosynostosis, photogrammetry, three-dimensional imaging

## Abstract

With increasing interest in 3D photogrammetry, diverse methods have been developed for craniofacial shape analysis in craniosynostosis patients. This review provides an overview of these methods and offers recommendations for future studies. A systematic literature search was used to identify publications on 3D photogrammetry analyses in craniosynostosis patients until August 2023. Inclusion criteria were original research reporting on 3D photogrammetry analyses in patients with craniosynostosis and written in English. Sixty-three publications that had reproducible methods for measuring cranial, forehead, or facial shape were included in the systematic review. Cranial shape changes were commonly assessed using heat maps and curvature analyses. Publications assessing the forehead utilized volumetric measurements, angles, ratios, and mirroring techniques. Mirroring techniques were frequently used to determine facial asymmetry. Although 3D photogrammetry shows promise, methods vary widely between standardized and less conventional measurements. A standardized protocol for the selection and documentation of landmarks, planes, and measurements across the cranium, forehead, and face is essential for consistent clinical and research applications.

Craniosynostosis is a condition involving premature fusion of one or multiple sutures. The compensatory growth of the suture perpendicular to the synostotic suture leads to deformity of the cranium, forehead, and face. The majority of the patients need surgical intervention to encourage normal skull growth and prevent the development of intracranial hypertension, and sensory and neurodevelopmental dysfunctions.^1–5^ The cranial shape and growth are often assessed with the use of cephalometric measurements on X-ray, computed tomography (CT) scans or manually measured. However, these approaches expose patients to harmful radiation, and with X-ray and manual measurements, there is potential for inaccuracies in representing the entire cranial shape as well as susceptibility to human error.

In recent years, there has been a surge of interest in utilizing 3-dimensional (3D) imaging techniques in clinical settings to monitor patients with craniosynostosis. In contrast to manual cephalometric measurements, 3D imaging methods are less susceptible to human error and also increase reproducibility. This is vital for preserving the integrity and reliability of measurements, ensuring that 3D photogrammetry assessments can be accurately and consistently replicated by other researchers.

3D photogrammetry is one of the imaging modalities of interest. Compared with traditional CT scans, 3D photogrammetry is easy to apply and noninvasive, requiring no radiation or possible sedation for a detailed image. Previous studies have indicated that routine CT scans carry small, but definite, risk of inducing malignancy and cognitive dysfunction.^6–8^ 3D photogrammetry is regarded as a safe and effective alternative to CT scans for evaluating craniofacial morphology during follow-up, particularly when no intracranial information is necessary.

The growing interest in 3D photogrammetry has led to the development of numerous methods and analyses for measuring cranial shape in patients with craniosynostosis. These are then implemented to assess surgical interventions, monitor long-term growth, and aid as diagnostic tools. However, the diverse range of methods introduces challenges in benchmarking and maintaining reproducibility. The aim of this review is to offer an extensive overview of the assortment of 3D cranial and facial shape analyses and the methodology utilized in the management of patients with craniosynostosis.

## METHODS

A systematic review was carried out according to the Preferred Reporting Items for Systematic Reviews and Meta-Analysis (PRISMA) statement^9,10^ (Supplemental Digital Content 1, http://links.lww.com/SCS/F938). In addition, this systematic review was registered prospectively in the International prospective register of systematic reviews (PROSPERO) with the following registration number: CRD42023409724.

### Selection Criteria

Inclusion criteria were original research reporting on 3D photogrammetry analyses in patients with craniosynostosis and written in English. Exclusion criteria were case reports, conference abstracts, editorials, letters, and notes, animal studies, studies including <10 patients with craniosynostosis, and publications describing 3D analysis on solely CT-scans, MRI, or ultrasound.

### Search

A systematic search of the literature was conducted to identify publications on 3D photogrammetry analyses in patients with craniosynostosis. The search was conducted within the following scientific databases: Medline, Embase, Web of Science Core Collection, and Cochrane Central Register of Controlled Trials. The databases were searched from inception until August 2023. The full search string of each database is included in Supplementary A, Supplemental Digital Content 2 (http://links.lww.com/SCS/F939). In addition, a manual search was performed of the reference lists of the included publications to identify additional relevant publications.

Two investigators (M.I.S.C.K. and P.A.E.T.) screened all publications independently on titles and abstracts for relevance. All documents that appeared to meet the selection criteria, as well as those that could not be adequately assessed from the information given, were retrieved and investigated as full text. After selection on title and abstract, full-text publications were assessed for eligibility based on inclusion and exclusion criteria.

### Data Collection and Analysis

The following data were extracted from the included publications: type of study, number of included patients, type of craniosynostosis, type of 3D imaging device used, type of 3D measurements, and related planes and landmarks. To guarantee the reproducibility of 3D photogrammetry analyses, it is crucial to precisely define and describe the landmarks and/or planes utilized for measurements. If a study failed to provide a comprehensive and clear description of the landmarks and/or planes used in their analysis, replicating the research and achieving similar results might prove challenging. Therefore, studies lacking a clear description of their landmarks and/or planes for a specific measurement were considered as non-reproducible and were excluded from our data analyses for that specific measurement. This approach is crucial for maintaining the integrity and reliability of measurements, as it ensures 3D photogrammetry measurements to be accurate and consistent for replication by other researchers.

### Study Quality and Risk of Bias

The Oxford Centre for Evidence-Based Medicine (CEBM) criteria were used to grade all studies on quality of evidence.

## RESULTS

### Study Selection

**FIGURE 1:**
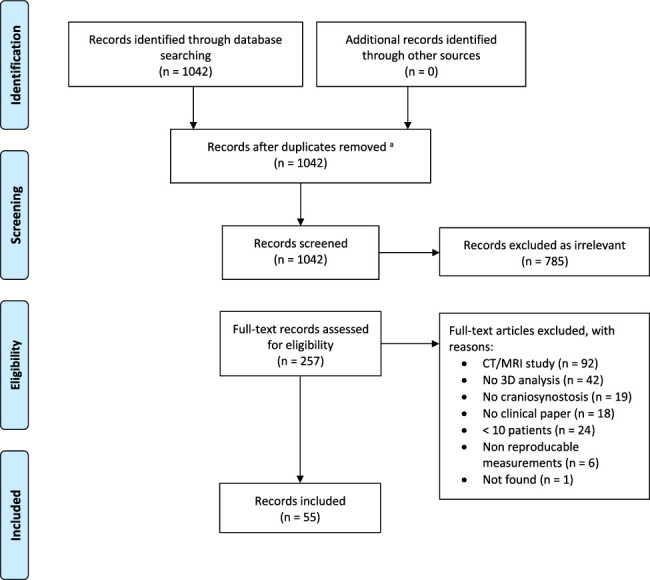
PRISMA diagram.

In total, 1083 publications were identified in the initial search and 1 publication after manual reference list search. Following the removal of duplicates, 1084 publications were screened for relevance on title and abstract. After the first screening, 818 publications were excluded, resulting in full-text assessment of eligibility in 266 publications. In total, 63 publications were included for qualitative analysis (Fig. 1).

### Study Characteristics

Of the 63 publications included, 55 publications had a retrospective study design, 1 had a prospective study design, and 7 were case-control studies. In addition to case-control studies, healthy controls were analyzed for comparison in 11 publications. Detailed characteristics are described in Supplemental Digital Content Table 1 (http://links.lww.com/SCS/F940). Several 3D imaging modalities were utilized, among which 44 publications used static imaging systems, 18 publications used dynamic imaging systems (including handheld devices, laserscanners, etc.), and 3 did not specify their used imaging modality. Patients with sagittal synostosis were included in 38 publications, metopic synostosis in 27 publications, unilateral/bilateral coronal synostosis in 10 publications, and lambdoid synostosis in 2 publications. A total of 3302 craniosynostosis patients and 1250 controls were analyzed in this review.

### Cranium

**FIGURE 2:**
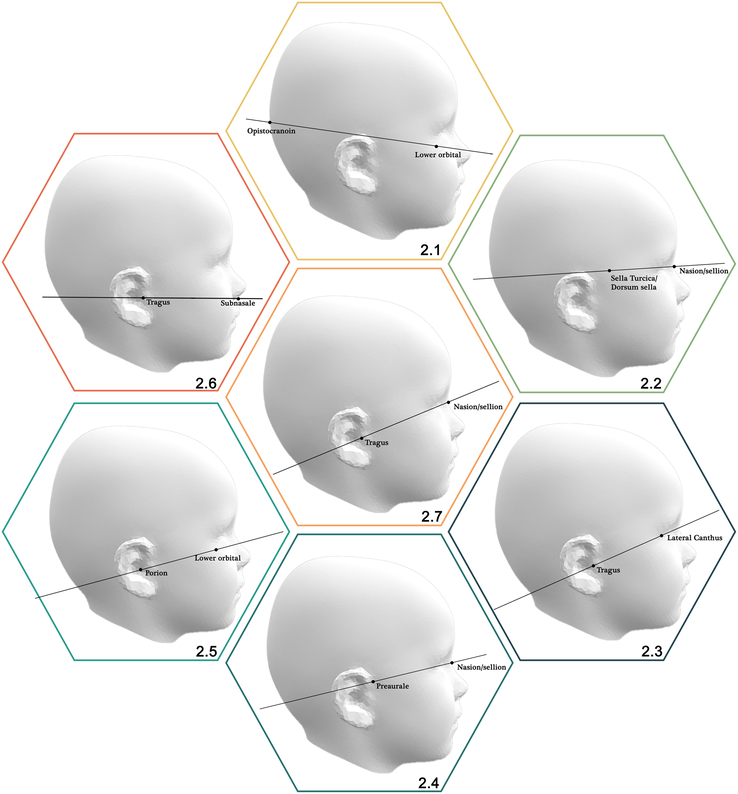
Base planes with corresponding landmarks 2.1. Opistocranion—lower orbital 2.2. Sella turcica/dorsum sella—nasion/sellion 2.3. Tragus—lateral canthus 2.4. Preaurale—nasion/sellion 2.5. Porion—lower orbital 2.6. Tragus—subnasale 2.7. Tragus—nasion/sellion.

In total, 52 publications described 3D measurements of the cranium, including volume measurement, width/length/height, cranial asymmetry, circumference, and cranial shape. Figure 2 provides a visual overview of all planes utilized in measurements of the cranium.

#### Volume Measurement

In total, 24 publications measured intracranial volume (ICV) in craniosynostosis patients. Of those 24 publications, 19 publications were considered as reproducible and are described in Supplemental Digital Content Table 2 (http://links.lww.com/SCS/F940). Fourteen publications defined ICV as the volume above a certain base plane.^11–28^ In 1 publication, the cranium above the base plane was divided into 10 proportionally spaced intervals, with level 0 at base plane and level 10 at the vertex.^14^ They defined the ICV as the volume between level 2 and level 8.

All publications used landmarks to create the base plane. Seven publications used both tragus and the nasion/sellion,^11,12,14,20,22,24,27^ and 4 publications used both tragus and the subnasale point.^19,25,26,29^ Three publications used the tragus and the lateral canthi as landmarks.^21,23,28^ Two publications used the sella turcica as a landmark, in which they used a new method (CCFP) based on CT scans to approximate the sella turcica.^15,16^ All landmarks, including other less frequently used landmarks are described in Supplemental Digital Content Table 2 (http://links.lww.com/SCS/F940).

McKay et al^23^ and Van Veelen et al^28^ have verified the validity of their method by conducting a comparison between the ICV obtained by 3D photogrammetry and CT scans, which is considered as the gold standard. Both showed high correlations (0.91 and >0.86, respectively) with a correction slope of 1.34 and 1.31 respectively.

#### Width, Length, and Height

Thirty-six publications measured width, length or height. Of these, 30 publications were considered as reproducible (Supplemental Digital Content Table 3, http://links.lww.com/SCS/F940). To measure width or length, there were 3 methods described, including measurements using a base plane in combination with a measurement plane,^11,14,18,20,22,24,25,29–32^ measurements using only a base plane^16,27,33–35,^ or measurements based on landmarks.^13,18,22,36–43^

The first method utilizes a base plane based on at least 3 landmarks (eg, both tragus and nasion/sellion or subnasale), after which parallel to the base plane, a measurement plane is created at a certain level, for example, at the maximum circumference, opisthocranion, or glabella. A frequently used level is at the maximum circumference.^30–32^ The maximum width and length are then obtained from the measurement plane. Alternatively, the level corresponding to maximum head length has been suggested for automated extraction of cephalic measurements. This method has shown to be more robust as it circumvents slices containing the ears, thus simplifying the measurement process.^12^

Another method uses landmarks to directly measure the width and length. The width is defined as the distance between euryon and euryon or tragus and tragus, in 8 publications^18,36–39,41–43^ and 2 publications,^13,22^ respectively. The length is defined as the distance between glabella and opisthocranion in all 10 publications.^13,18,36–43^

The first 2 methods use a base plane. For this base plane, the most commonly used landmarks are the left and right tragus and the nasion/sellion, which is used in 9 publications,^11,14,20,22,24,27,33–35^ or the left and right tragus and the subnasale, which is used in 2 publications.^25,29^ With the maximum width and length, the cephalic index can be calculated, which has been performed in 23 publications.^11,13,14,16,18,22,24,25,27,29–39,41–43^

Height was measured in several ways across different studies. It has been defined as the distance between the vertex and the porion,^41,44^ and alternatively as the distance between the vertex and the nasion.^21,39^ Another approach considered the height as the distance between the vertex and the midpoint between the 2 tragi.^13^

#### Cranial Asymmetry

Cranial asymmetry can be measured using diagonal measurements to calculate Cranial Vault Asymmetry Index (CVAI) or the diagonal difference (DD). Eleven publications measured diagonals, of which 6 publications were considered as reproducible and are described in Supplemental Digital Content Table 4, http://links.lww.com/SCS/F940. These diagonals were obtained from a measurement plane, which was created by landmarks of the glabella and opisthocranion^18^ or parallel to the base plane at a certain level.^14,17,22,30,45^ The base plane was created with landmarks of both tragus and sellion/nasion in 3 publications.^14,22,45^ The other 2 publications used both tragus and both exocanthions,^30^ or both preaurarales and the sellion.^17^ The measurement plane was created, as being parallel to the base plane at a certain level, but the corresponding level varied between the publications. The diagonals were calculated at a 30° or 60° angle from the midline.

Another method for determining cranial asymmetry is by measuring the ratio between quadrant volumes. Eight publications used the quadrant method for their ICV and/or asymmetry ratios.^14,15,17–19,22,24,29^ All articles used the same method but utilized different landmarks, as described in Supplemental Digital Content Table 2 (http://links.lww.com/SCS/F940).

The ICV can be divided into 4 quadrants (anterior left, anterior right, posterior left, posterior right), determined by the intersection of x-axis, y-axis, and z-axis. With the y-axis as a vertical plane through the nasion/sellion or subnasale and the midpoint (being an intersection of the tragus or preaurales connection line with nasion/sellion or subnasale), with the x-axis perpendicular to the y-axis and through the midpoint, and the z-axis as perpendicular to the x-axis. The volume of the 4 quadrants created with this method are then calculated and used for symmetry ratios such as the anterior/posterior asymmetry index (ACAI/PCAI), anterior/posterior asymmetry ratio (ASR/PSR), or anteroposterior volume ratio (APVR).

#### Circumference

The cranial circumference can be measured by the head circumference, coronal circumference, or sagittal circumference. In total, 18 publications measured a cranial circumference, of which 14 publications were considered as reproducible and are described in Supplemental Digital Content Table 5, http://links.lww.com/SCS/F940.

Thirteen publications measured the head circumference.^11,12,14,16,22,24,25,30–32,40,41,45^ Of these 13 publications, 10 used a measurement plane parallel to the base plane.^11,12,14,22,24,25,30–32,45^ The base plane was mostly created using the landmarks left and right tragus and nasion/sellion.^11,12,14,22,24,45^ In addition, other publications used the Frankfort horizontal plane,^32^ a plane through the left and right tragus, subnasale,^25^ left and right exocanthions,^30^ or the nasion and clinoid processes dorsum sella.^31^ The measurement plane was then created parallel to the base plane at a certain level, with the majority of the publications at the level of maximum circumference.^11,12,30–32^

The coronal circumference was measured in 2 publications,^22,38^ and was defined as the circumference over the top of the head from left to right ear. The sagittal circumference was measured in 1 publication, and defined as the circumference over the top of the sagittal plane starting at plane 0 at nasion.

#### Cranial Shape

In total, 23 publications have tried to visualize the cranial shape in craniosynostosis patients (Supplemental Digital Content Table 6, http://links.lww.com/SCS/F940). Fifteen publications have used heat maps to show changes in distances between 2 groups.^15,16,27,30,31,34,42,44,46–52^ These groups can be a preoperative and postoperative group to show relative change, or a patient population and normal population to show absolute differences. Curvature analysis specifically visualizes the local curvature, which is performed in 3 publications.

Two publications use sinusoidal curves to show the contour of the cranial shape at an axial slice.^53,54^ Based on the sinusoidal curves, patterns for types of craniosynostosis are distinguished.

Four studies employ principal component analysis (PCA), a statistical procedure that identifies and quantifies shape variations from a standard or ‘normal’ population.^33,55–57^ By concentrating on the most significant variations, PCA enables the differentiation of distinct types of craniosynostosis. Furthermore, it helps to identify the characteristic components or features associated with each specific type of craniosynostosis.

One publication used an anteroposterior area ratio to measure the differential growth in scalp surface area between front and back half of neurocranium.^17^

### Forehead

**FIGURE 3:**
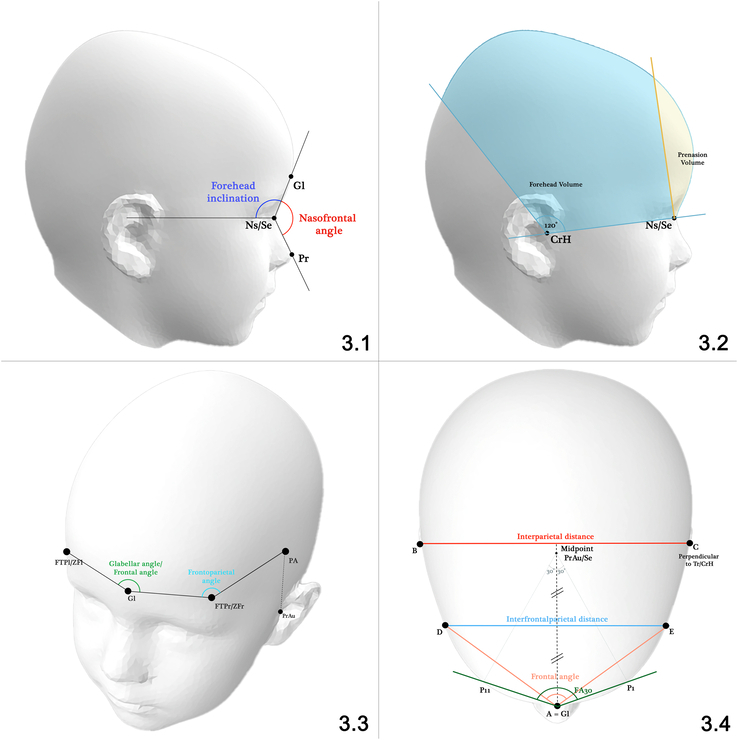
Forehead measurements. 3.1. Frontal prominence in angles; 3.2. Forehead volume; 3.3. Frontal angle and Frontoparietal angle; 3.4. Frontal angle and frontal width. Ns/Se, nasion/sellion; Gl, glabella; Pr, prenasale; CrH, crus helix; FTPl/ZFl, frontotemporal point left/zygomaticofrontal left; PA, parietal point; PrAu, preaurale; FA30, Frontal angle 30; A/B/C/D/E, corresponding to Rodriguez-Florez^52^.

In total, 17 publications described measurements on 3D photogrammetry of the forehead (Supplemental Digital Content Table 7, http://links.lww.com/SCS/F940). These publications have tried to measure the forehead prominence, frontal width, forehead wedging, and temporal hollowing or to visualize the total frontal shape. Figure 3 provides a visual overview of all measurements utilized to describe forehead shape.

#### Frontal Prominence

Five publications focus on the frontal prominence by measuring either a volume or an angle.^20,21,30,52,58^ Two publications described volume measurements, including 1 publication measuring forehead volume^52^ and 1 publication prenasion volume.^58^ In addition, in the earlier described section on cranial volume, 3 publications described forehead volume in addition to other volumetric measurements. Four publications described the measurement of angles, including 2 publications measuring the forehead inclination^21,58^ and 2 publications measuring the nasofrontal angle.^20,30^ The forehead inclination describes the angle between the prominence of the forehead and the Frankfort horizontal plane, while the nasofrontal angle focuses on the angle between the prominence of the forehead and the nose.

#### Fontal Width

Frontal width was described in 6 publications^20,37,40,52,59,60^ of which 4 publications described frontotemporal distance.^37,40,59,60^ In addition, 2 publications described the interfrontoparietal–interparietal ratio, which focuses on the relative width of the forehead compared with the parietal width.^20,52^

#### Frontal Wedging and Temporal Hollowing

Eight publications focused on frontal wedging, including measurement of the glabellar angle, FA30, and frontal angle.^17,20,37,38,52,59,61,62^ All measurements used the glabella as the corner point of their angle, while the rays differ between methods (Fig. 3).

Three publications described temporal hollowing, all utilizing the same technique, by measuring the frontoparietal angle.^37,38,62^

#### Frontal Shape

Four publications described frontal shape,^45,63–65^ of which 2 publications described the distances between the origin and predetermined points on the forehead,^63,64^ 1 publication described a mirroring technique,^65^ and 1 publication measured the area under the sinusoidal curve of the forehead.^45^

In the earlier described section cranial shape, 2 of the 3 publications that used curvature analysis focus additionally on the forehead and temporal areas (Supplemental Digital Content Table 6, http://links.lww.com/SCS/F940).^48,66^

### Face

In total, 8 publications measured facial asymmetry on 3D photogrammetry (Supplemental Digital Content Table 8, http://links.lww.com/SCS/F940).^30,65,67–72^ Four publications described facial asymmetry/symmetry by mirroring 1 side of the face using a best-fit technique and measuring the distances between corresponding points of the 2 sides.^67,69–71^ Besides the asymmetry analysis of the whole face, 2 publications analyzed 6 subregions,^69,70^ 1 publication analyzed 5 subregions,^71^ and 1 publications analyzed facial vertical thirds and areas of known dysmophology.^67^ In 3 publications, facial asymmetry was described by calculating the facial midline deviation and nasal deviation angle.^65,68,72^ One publication measured the exocanthion index to describe facial asymmetry.^30^

In addition, 6 publications used anthropometric measurements of the face, which are based on predetermined landmarks (see Supplemental Digital Content Table 6, http://links.lww.com/SCS/F940).^20,59,65,68,71,72^

## DISCUSSION

This study is the first comprehensive review that presents an overview of the methodology used to measure shape of the cranium, forehead, and face on 3D photogrammetry in craniosynostosis patients. This review shows a wide range of methodologies for processing and measuring cranial and facial shape in children with craniosynostosis. Landmarks such as the tragus, subnasale, and nasion/sellion are commonly utilized for alignment and positioning of 3D images. Based on these landmarks, a base plane can be established, and if needed, a measurement plane is created which is usually parallel to the base plane. The level on which the measurement plane is created is often at maximum circumference. Assessments of base or measurement plane-derived parameters like volume, width, length, height, circumference, and diagonal distances play a crucial role in cranial studies. However, for a more comprehensive evaluation of cranial shape, heatmaps, and curvature analyses are frequently employed. These techniques offer a more detailed insight into the growth dynamics, shape transformations, volume alterations, and curvature evolution over time or in comparison with the general population. We show that for the assessment of the forehead, a wide variety of methodologies is found, including volumetric measurements, angles, ratios, and mirroring techniques. To assess the face, the most commonly utilized methodology was mirroring 1 side of the face and measuring the distances between the 2 sides. In addition, the application of angles, indices, and anthropometric measurements are applied to describe the face.

The majority of the studies have used standardized measurements extracted from 3D photogrammetry data, including ICV, width, length, height, diagonals, and circumference, as their outcome parameters. The outcome of these standardized measurements has the advantage of being used in 2 ways: they enable the assessment of relative differences in individual patients through pre- and postoperative evaluation, as well as the determination of absolute values useful for benchmarking. The reliability of these outcomes can be impacted by several factors, leading to increased variability and uncertainty. The use of different methods, variations in absolute values caused by utilization of different imaging modalities as well as inter- and intra-rater variability can all contribute to this increase in uncertainty and variability.

### Difference in Methods

The ever-increasing number of methods used to process and analyze (3D) data show the development of the research field, but introduce at the same time variability that make outcome interpretation and comparison across studies challenging, in particular, when these methodologies are not well described and therefore not reproducible. For example, the majority of the articles define ICV as the volume above the base plane; however, various landmarks are used to create the base plane across publications. Of the 15 articles measuring volume, there are 6 different base planes used.

After defining a base plane, a measurement plane can be created which is often parallel to the base plane. The level at which the measurement plane is created also varies between studies. The majority of the studies placed their measurement plane at the level of maximum circumference. However, for some measurements such as the width, length, and height, a base plane is not always used. Instead, 2 landmarks are used to directly measure the distance between landmarks.

A complex question that remains is to what extent differences in methodology can potentially affect the results and conclusions of a study. When studies describe changes over time within the same patient/group, differences in methodology are less problematic, provided they are well described and reproducible. However, if the outcomes are presented as absolute values for the purpose of comparison with other studies/centers, variations in methodologies can undeniably influence the final conclusions. Al-Shaqsi et al (13) focused on whether the cranial length measurement’s methodology would impact the Cranial Index (CI). They compared 2 different approaches: the traditional CI method, where cranial length was calculated based on the distance between the glabella and the opisthocranion, and a second method which measured the maximal cranial length from the anterior point to the opisthocranion on a horizontal plane. They concluded that different methods can result in different absolute values. If studies desire to use absolute values as their outcome, they should be attentive to use a control/comparison group which was measured using the same method, or use a validated method.

### 3D Photogrammetry Compared with the Gold Standard

Measurements on 3D photogrammetry are based on exterior soft tissue of the cranium and face. However, if a measurement does not rely on the exterior soft tissue of the cranium, the outcome of a measurement based on 3D photogrammetry cannot be seen as a direct or exact comparison, as is the case with ICV. These measurements are often validated on other imaging modalities such as CT scans, MRI, etc. When comparing measurements on 3D photogrammetry and other imaging modalities, absolute values might differ.

### Interrater and Intrarater Reliability

In the context of 3D photogrammetry, multiple measurements have been confirmed to be reproducible. For Cranial volume, the methods by^23,28^ have been proven to have a high interrater reliability of 0.98 and 0.99, respectively^14^ showed a high intra-rater reliability of 0.995. Other measurements such as width, length, diagonals, circumference, and the prenasion volume have also been proven to have a high intrarater reliability.^14,39,58^ Facial distances have also been proven to be reliable measurements utilized for 3D photogrammetry.^59,73^

### Landmarks

Landmarks are commonly used in the analysis of 3D photogrammetry. Landmarks serve various purposes, including image alignment, plane definition, or direct anthropometric measurements.

One of the challenges in using 3D photogrammetry is finding a reliable and consistent landmark for different images over time.^74^ Landmarks with well-defined borders or edges showed higher degrees of reproducibility than those placed on gently curving slopes.^75^

The difference in hard and soft tissue landmarks can influence the reliability on 3D photogrammetry. For example, soft tissue landmarks, such as the lateral canthi, are more visible on the 3D photogrammetry and less visible on a CT scan. Landmarks based on bony structures can be challenging in 3D photogrammetry, as bony structures are not visible or palpable.^76^ Without the use of CT-based reference frames for 3D photogrammetry analysis, the identification of bony structures as landmarks needs to be reviewed.

Among all included studies, 12 publications used landmarks based on bony structures for the analysis of 3D images, such as the nasion, lower orbital rim (in case of the Frankfort horizontal plane), sella turcica, or clinoid processes dorsum sella. Of the included studies, de Jong et al and Tu et al^15,31^ have approximated the bony landmarks using CT scans, de Jong et al^15^ utilized a CT-based program to define the sella turcica as a landmark on 3D photogrammetry, and Tu et al^31^ created a reference plane and landmarks based on CT scans.

Although there is generally a high level of precision and reproducibility observed for landmarks overall, it is important to note that different landmarks exhibit substantial variation in their degree of reproducibility.^75,77^ In the literature, the soft gonion was more often found to have poor reproducibility and precision.^75,77,78^ A potential explanation for this discrepancy could be that some landmarks are generally identified by examiners in clinical practice through palpation of underlying bony structures.^77^ In addition, the limited reproducibility may be influenced by lack of color contrast and the presence of shadows.^75^

Landmarks in the midline are often the nasion/sellion or subnasale. Among all included publications the nasion is used as a landmark in 10 publications, sellion in 5, and subnasale in 4. The nasion is a complex landmark for analysis over time in craniosynostosis as some surgical techniques affect the nasion.^79^ It should be noted that the landmark placement of the nasion is often incorrect.^80^ Determining the nasion, which is the midpoint of the nasofrontal suture, introduces challenges in 3D photogrammetry as it involves locating a bony structure. Another important landmark in this region is the sellion, defined as the deepest point of the nasofrontal region. In their study, Bahsi et al^80^ demonstrated that in approximately 59% of cases, the nasion and sellion were identified at the same location. However, in 41% of cases, the sellion was on average situated 4.53 ± 0.95 mm below the nasion. This finding challenges the common belief of many that the nasion corresponds to the point of greatest concavity in the midline between the forehead and nose. Instead, the nasion is often positioned above the sellion, making it a challenging landmark to accurately place in the context of 3D photogrammetry. The reproducibility of the nasion has a wide variety in intra-rater and inter-rater reliability, possibly caused by differences in definition and the difficulty in positioning of the head for landmark placement.^75,81–83^ Therefore, it is advisable to use the sellion over the nasion as a landmark for 3D photogrammetry. The precision in placement of the subnasale landmark has been prone to involuntary facial expression. The nasolabial and mouth region has been prone to facial expression.^84^ Masoud et al^85^ reported difficulties in locating the subnasale due to the variation in lip posture, as well as nose and lip morphology. When comparing the precision of anatomical landmarks, the nasion/sellion has been proven to be easily located with smaller margin errors, in contrast to the subnasale.^86^

For lateral landmarks, the endocanthions, exocanthions, and tragus are often used. Reproducibility and precision of these landmarks are also discussed in the literature but described with wide variation for both endocanthion and exocanthion^75–77,81,87–89^ and the tragus.^75,76,88,90,91^ Subtle movement or blinking can affect the resolution around the eyes.^92^ Additionally, the periocular region differs between ethnicities.^93,94^ The placement of landmarks positioned on the ear is difficult because of artifacts and shadows due to hair.^88^ If possible, tight hair caps can be used to minimize the artifacts due to hair.

Identifying lateral landmarks can pose a challenge in patients with unicoronal or unilambdoid synostosis, given their cranial asymmetry, which causes their eyes and ears to be positioned asymmetrically.

Bony structures also have a crucial role in the definition of planes. One of the most commonly used planes, the Frankfort Horizontal plane, is defined by bony structures.^95^ The Frankfort Horizontal plane passes through the porion, defined as the upper rim of the external acoustic meatus, and the orbitale, defined as the lowest point of the orbital rim. Among all included studies, 6^21,32,38,58,59,62^ described the use of the Frankfort horizontal plane. In the context of CT scans and X-rays, the Frankfort Horizontal plane is commonly used and easily identifiable; however, in 3D photogrammetry, the definition of the bony structures for this plane comes with challenges.^88^ Of all included articles, none described a description of the methodology employed to define the Frankfort Horizontal plane or the specific placement of corresponding landmarks.

### Type 3D Imaging Modalities

There is a wide variety of 3D photogrammetry devices. The difference in imaging modalities is primarily based on the difference in use. The majority of the studies use a setup that consists of multiple optical cameras to ensure a fast and accurate 3D image. These static devices are validated and reliable imaging systems.^77,96^

Laser scanners and handheld scanners are also validated devices.^97–99^ These systems are designed to take sequential images and/or must be moved around the object and are therefore prone to movement artifacts,^100,101^ especially in the orbital and nasolabial areas.^102^ Patients should be able to hold still for multiple seconds as the scanner rotates around the face. Additionally, differences in imaging devices can lead to different landmark placement of the otobasion inferior, exocanthion, and gnathion.^92^ This can be due to the capture field and/or movement during imaging. Gibelli et al^103^ concluded a high reliability in laser scanners for linear distances, angles, and surface area measurements, but discourages volume assessment. The advantage of handheld scanners is the portability of the device. These devices can be used in different settings, whereas large static photogrammetry setups require a dedicated room and need to be regularly calibrated. Lastly, the cost of a portable scanner is lower, compared to a static device.^92^

### Limitations Own Research

Like any systematic review, ours is not without limitations. First, the inclusion of studies with a wide range of sample sizes introduced variability. Studies with fewer than 10 patients were excluded, resulting in the exclusion of several publications on 3D analyses in craniosynostosis with very small samples. Second, this systematic review focused solely on 3D analysis techniques applied on patients with craniosynostosis. Therefore, other techniques employed in studies with different patient populations were not considered.

## RECOMMENDATIONS AND CONCLUSION

3D photogrammetry is promising and widely used to measure and visualize cranial shape. The past literature has shown a wide variety of methods for standardized and unstandardized measurements, and the majority of the measurements are unable to capture the complex craniofacial morphology and all its intricacies completely.

Based on our current study, preliminary recommendations on 3D photogrammetry analysis can be made. To address issues with variability and reliability, we recommend the use of the sellion as the landmark in the midline. This choice has advantages because the sellion is less susceptible to facial expression, as is the subnasale. Moreover, the subnasale may deviate from the midline in cases of nasal or septum deviation. Furthermore, the sellion is preferred over the nasion, as the nasion is a bony structure, which presents challenges in its precise determination using 3D imaging techniques. As for the lateral landmarks, we recommend incorporating the tragus.

Especially in young children, since they tend to be more active it is challenging for them to remain seated for extended periods. Finally, researchers should be aware of the wide range of methodologies for analyzing and measuring cranial and facial shape. Currently, analyses that allow for visualizing and measuring the complete cranial or shape are still lacking. Due to the variety in methods, benchmarking results remain complicated. Therefore, we advocate a standardized protocol to process and measure cranial and facial shape in this growing field. Global collaboration is essential to be able to facilitate larger studies and help with the pursuit of reaching consensus on treatment options and understanding of craniosynostosis, which is the objective of the ERN CRANIO 3D working group.

## Supplementary Material

SUPPLEMENTARY MATERIAL
