# Supplementary material for: 3D Analysis of the Cranial and Facial Shape in Craniosynostosis Patients: A Systematic Review
Source: J Craniofac Surg. 2024 Mar 18;35(3):813–21. doi: 10.1097/SCS.0000000000010071 (PMC11045556; doi:10.1097/SCS.0000000000010071)
Supplement: SUPPLEMENTARY MATERIAL [file scs-35-0813-s002.docx]

**Supplementary A -** Search

**Medline 733**

(exp Craniosynostoses / OR (craniosynostos* OR craniosteno* OR stenocephal* OR scaphocephal* OR trigonocephal* OR oxycephal* OR acrocephalosyndactyl* OR apert OR chotzen OR pfeiffer OR ((craniofacial* OR crani* OR saggit* OR metopic* OR unicoronal* OR coronal* OR sagittal* OR lambdoid*) ADJ6 (synostos* OR premature-closure*)) OR acrocephal* OR hypsicephal* OR oxycephalia OR pyrgocephal* OR steeple-head* OR steeple-skull* OR tower-head* OR tower-skull* OR turricephal*).ab,ti,kw.) AND (Imaging, Three-Dimensional / OR ((3d OR 3-D OR three-dimension* OR 3-dimension*) ADJ10 (imag* OR ct OR photo* OR quantificat* OR morphomet* OR analy* OR tomogra* OR scan* OR cephalometr* OR volum*)).ab,ti,kw. OR ((3d OR 3-D OR three-dimension* OR 3-dimension*) NOT print*).ti.) NOT (exp animals/ NOT humans/) NOT (news OR congres* OR abstract* OR book* OR chapter* OR dissertation abstract*).pt. AND english.la.

**Embase 749**

('craniofacial synostosis'/exp OR (craniosynostos* OR craniosteno* OR stenocephal* OR scaphocephal* OR trigonocephal* OR oxycephal* OR acrocephalosyndactyl* OR apert OR chotzen OR pfeiffer OR ((craniofacial* OR crani* OR saggit* OR metopic* OR unicoronal* OR coronal* OR sagittal* OR lambdoid*) NEAR/6 (synostos* OR premature-closure*)) OR acrocephal* OR hypsicephal* OR oxycephalia OR pyrgocephal* OR steeple-head* OR steeple-skull* OR tower-head* OR tower-skull* OR turricephal*):ab,ti,kw) AND ('three-dimensional imaging'/de OR ((3d OR 3-D OR three-dimension* OR 3-dimension*) NEAR/10 (imag* OR ct OR photo* OR quantificat* OR morphomet* OR analy* OR tomogra* OR scan* OR cephalometr* OR volum*)):Ab,ti,kw OR ((3d OR 3-D OR three-dimension* OR 3-dimension*) NOT print*):ti) NOT ([animals]/lim NOT [humans]/lim) NOT ([conference abstract]/lim) AND [english]/lim

**Cochrane 0**

((craniosynostos* OR craniosteno* OR stenocephal* OR scaphocephal* OR trigonocephal* OR oxycephal* OR acrocephalosyndactyl* OR apert OR chotzen OR pfeiffer OR ((craniofacial* OR crani* OR saggit* OR metopic* OR unicoronal* OR coronal* OR sagittal* OR lambdoid*) NEAR/6 (synostos* OR premature-closure*)) OR acrocephal* OR hypsicephal* OR oxycephalia OR pyrgocephal* OR steeple-head* OR steeple-skull* OR tower-head* OR tower-skull* OR turricephal*):ab,ti) AND (3d OR "3-D" OR three-dimension* OR "3-dimension*"):ab,ti

**Web of science 595**

TS=(((craniosynostos* OR craniosteno* OR stenocephal* OR scaphocephal* OR trigonocephal* OR oxycephal* OR acrocephalosyndactyl* OR apert OR chotzen OR pfeiffer OR ((craniofacial* OR crani* OR saggit* OR metopic* OR unicoronal* OR coronal* OR sagittal* OR lambdoid*) NEAR/5 (synostos* OR premature-closure*)) OR acrocephal* OR hypsicephal* OR oxycephalia OR pyrgocephal* OR steeple-head* OR steeple-skull* OR tower-head* OR tower-skull* OR turricephal*)) AND (((3d OR 3-D OR three-dimension* OR 3-dimension*) NEAR/10 (imag* OR ct OR photo* OR quantificat* OR morphomet* OR analy* OR tomogra* OR scan* OR cephalometr* OR volum*)))) NOT DT=(Meeting Abstract OR Meeting Summary) AND LA=(English)
