# Supplementary material for: 3D Analysis of the Cranial and Facial Shape in Craniosynostosis Patients: A Systematic Review
Source: J Craniofac Surg. 2024 Mar 18;35(3):813–21. doi: 10.1097/SCS.0000000000010071 (PMC11045556; doi:10.1097/SCS.0000000000010071)
Supplement: SUPPLEMENTARY MATERIAL [file scs-35-0813-s003.docx]

**Tables 3D analysis in craniosynostosis: systematic review**

**Supplementary** Table 1. Study characteristics

| **Author** | **Year** | **CEBM level of evidence** | **Study design** | **Sample size** | **Patient characteristics** | **Procedure** | **3D imaging modality** | **Aim** |
| --- | --- | --- | --- | --- | --- | --- | --- | --- |
| Al-Shaqsi et al. (1) | 2018 | 4 | Retrospective cohort | 14 | SS | Unoperated | 3dMD (Atlanta, GA) | Assess change head shape, volume, CI over time |
| Al-Shaqsi et al.(2) | 2021 | 4 | Retrospective cohort | 55 | SS | Open total cranial vault reconstruction vs endoscopic suturectomy | 3dMD (Atlanta, GA) | Compare morphological outcome endoscopic vs open reconstruction |
| Applegren et al.(3) | 2018 | 4 | Retrospective cohort | 203  Cases: 87  Controls: 116 | MS^a^ |  | STARscanner (Orthomerica, Orlando, FL) | Assess head shape and anterior cranial volume |
| Badiee et al.(4) | 2022 | 4 | Retrospective cohort | 35 | MS | SCOT or OCVR | Canfield Vectra H2 (post-op) and CT or laser scans (pre-op) | Compare long term outcomes of SCOT vs OCVR |
| Borghi(5) | 2022 | 4 | case-control | 24  Cases: 18  Controls: 6 | SS | SAC | Rodin4D surface scanner or CT | Assess perception of craniofacial deformity, geometric head features, and head shape |
| Cho(6) | 2018 | 4 | Retrospective cohort | 43 | MS | Surgical & non-surgical | 3dMD (Atlanta, GA) and CT | Evaluate and quantify surgeons’ thresholds for operative intervention |
| Chou(7) | 2017 | 4 | Retrospective cohort | 21 | SS | sagittal strip craniectomy | STARscanner | Assess surgical outcomes |
| de Jong(8) | 2017 | 4 | Retrospective cohort | 86 | MS | ESC with helmet therapy | 3dMD head system | Post-op evaluation |
| de Jong(9) | 2020 | 3 | Retrospective cohort | 196  Cases: 143 Controls: 53 | SS, MS, UCS |  | 3dMDCranial (Atlanta, GA) | Create a deep learning network to differentiate between craniosynostosis |
| Elawadly(10) | 2023 | 4 | Retrospective cohort | 13  Cases: 7  Controls:6 | UCS | ESC with postoperative helmeting | 3dMDHead system, m4D scan Rodin, or TechMed3D Body Scanner | Assess surgical outcome |
| Elawadly(11) | 2022 | 4 | Retrospective cohort | 6 (+15 controls) | MS | ESC with postoperative helmet orthosis therapy | 3dMDHead system, m4D scan Rodin, or TechMed3D Body Scanne | Characterize trigonocephaly and quantified the morphological outcomes |
| Freudlsperger (12) | 2015 | 4 | Retrospective cohort | 18 | MS | FOA | VECTRA-360-nine-pod system (Canfield Science, Fairfield, NJ, USA) | Evaluate intracranial volume |
| Gabrick(13) | 2020 | 3 | Retrospective cohort | 21 | UCS |  | VECTRA H-1 portable 3D camera | Assess long-term facial asymmetry |
| Harrison(14) | 2023 | 3 | Retrospective cohort | Cases: 50  Controls: 50 | SS | ESC with post-operative helmet therapy | 3dMD (Atlanta, GA) | Quantify forehead convexity and temporal convexity |
| Heutinck(15) | 2021 |  | Retrospective cohort | Cases: 94  Controls: 65 | SS |  | Handheld scanner; M4D Scanner, Rodin4D or CT | Create a population-based 3D head shape model for the 0 to 2-year-old normal population |
| Klausing(16) | 2019 |  | prospective study | Cases: 80  Controls: 49 | MS, SS, CS |  | 3D surface scan (3DShape, Erlangen, Germany) | Assess morphology in synostosis |
| Kronig(17) | 2022 | 3 | Retrospective cohort | 32 | UCS and positional posterior plagiocephaly |  | 3dMD (Atlanta, GA) | To quantificate severity |
| Kronig (18) | 2023 | 4 | Retrospective cohort | 25 | SS, MS, CS |  | 3dMD (Atlanta, GA) | To quantificate severity |
| Kuehle (19) | 2022 | 4 | Retrospective cohort | 57 (+ 296 controls) | MS | FOR | VECTRA-360-nine-pod system (Canfield Science, Fairfield, NJ, USA) | Asses head shapes and cephalometric measurements |
| Le (20) | 2014 | 4 | case-control | 40  Cases: 20  Controls: 20 | SS |  | 4-pod camera system (3dMD, Atlanta, GA) | Evaluate the long-term cephalometric results |
| Linden (21) | 2019 | 4 | Retrospective cohort | 13 (+ matched healthy controls) | MS | Anterior Cranial Vault remodeling | Canfield  Vectra stereophotogrammetry system | evaluating the postoperative morphologic changes |
| Linz (22) | 2014 | 4 | Retrospective cohort | 20 | SS | craniectomy, active tilting of the forehead, and bitemporal greenstick fracturing | Infant recordings 3dMD®, Atlanta, GA, USA | evaluated a standardized operative procedure |
| Martini (23) | 2015 | 3 | Retrospective cohort | 13 | MS, CS | FOA | 3D light optical scanner | evaluate the frontoorbital region after cranioplasty with FOA |
| Martini(24) | 2017 | 3 | Retrospective cohort | 28 | MS, CS | FOA | 3D light optical scanner | evaluation of symmetry and its changes in the forehead during FOA |
| McKay(25) | 2010 | 3 | Retrospective cohort | 60 | SS |  | 3D photogrammetry 3dMD (Atlanta, GA) + 3D CT | to correlate cranial volume measured from 3D photograph and CT scan |
| Mertens (26) | 2017 | 3 | Retrospective cohort | 85 | SS | ESC, MMP | Canfield VECTRA-360-nine-pod system | to compare the outcome in ICV and CI between ESC  and MMP techniques |
| Meulstee (27) | 2017 | 3 | Retrospective cohort | 40 | SS, MS |  | 3dMDCranial 3DMD, Atlanta, USA, Five-pod | to present a  new method for the objective evaluation  of the 3D morphology of the cranial shape |
| Oh (28) | 2008 | 3 | Retrospective cohort | 30 | UCS | Bilateral FOA | 3dMDface system | describe facial asymmetry in adolescents and adults with corrected UCS |
| Owall (29) | 2016 | 4 | Case-control | 44  Cases: 22  Controls: 22 | UCS | bilateral craniotomy of the frontal bone with unilateral  orbital rim advancement | 3dMDtrio system | to assess facial soft-tissue asymmetry in UCS |
| Owall (30) | 2021 | 3 | Retrospective cohort | 21 | UCS | Unoperated | 3dMDtrio system | compare the degree facial asymmetry in infants with NS-UCS and MS-UCS |
| Owall (31) | 2019 | 3 | case-control | 33  Cases: 11  Controls: 22 | UCS | bilateral craniotomy of the frontal bone and unilateral supraorbital rim advancement | 3dMDtrio system | correlation between the degree of facial asymmetry pre- and postsurgically |
| Pfaff(32) | 2023 | 3 | Retrospective cohort | 41 | MS | FOA | 3dMD + 3D CT | Evaluate fronto-orbital “overcorrection” approach in trigonocephaly |
| Pickersgill (33) | 2018 | 3 | Retrospective cohort | 41 | SS | Endoscopic repair | 4-pod 3dMD + 3D CT | to determine CI of patients with endoscope-assisted  strip craniectomy and postoperative helmet therapy |
| Porras (34) | 2019 | 3 | Retrospective cohort | 116 cases;  201 control | SS, MS, CS, multisuture |  | 3dMDhead System + 3D CT | present a novel methodology to quantitatively evaluate the head shape from 3D photography |
| Pressler (35) | 2021 | 3 | Retrospective cohort | 34 | MS | Open Vault reconstruction | Unspecified + 3D CT | demonstrate the 3D head after LISC or OCVR intervention for MCS |
| Ramamurthi (36) | 2021 | 3 | Retrospective cohort | 24 | SS | Helmet | 3dMD  Vultus   + 3D CT | to compare the outcomes from a novel helmet design protocol compared to a traditional helmet design protocol |
| Rodriguez-Florez (37) | 2020 | 3 | Retrospective cohort | 30 | SS | SAC | Handheld scanner; M4D Scanner, Rodin4D | Assess 3D headshape change after calvarial remodelling |
| Rodriguez-Florez (38) | 2017 | 3 | Retrospective cohort | 18 | SS | SAC | Handheld scanner; M4D Scanner, Rodin4D | investigate microstructure of cranial bone in SS undergoing SAC |
| Rodriguez-Florez(39) | 2017 | 3 | Retrospective cohort | 10 | MS | FOA | Handheld scanner; M4D Scanner, Rodin4D | compare 3D head shape of patients before and after FOR |
| Sakar (40) | 2022 | 3 | Retrospective cohort | 24 | MS | ESC | STARscanner | Propose a new skull outline based method to objectify quantify complex 3D skull shapes |
| Schaufelberger (41) | 2022 | 3 | Retrospective cohort | Cases: 189  Controls: 178 | UCS, MS, SS |  | Canfield VECTRA-360-nine-pod system | Construct a classification pipeline for craniosynostosis |
| Schulz (42) | 2021 | 4 | Case-control | 128  Cases: 80  Controls: 48 | SS, MS | biparietal craniectomy/FOR  Endoscopically assisted strip craniectomy | Vectra M5-360 Head  System, Canfield Scientific | Evaluation of morphometric outcomes of Single suture craniosynostosis |
| Seeberger (43) | 2016 | 3 | Retrospective cohort | 71 | SS | Untreated (pre-operative) | Canfield VECTRA-360-  nine-pod system | preoperative ICV of SS patients |
| Seruya (44) | 2014 | 3 | Retrospective cohort | 31 | MS | FOA | 3dMD, Atlanta | Compare cranial growth for three patterns of FOA |
| Sharma (45) | 2018 | 3 | Retrospective cohort | 11 | SS | Melbourne technique | Handheld scanner (M4D scanner, Rodin4D) and 3D CT | Report results on melbourne technique |
| Svalina (46) | 2022 | 4 | case control | 114  Cases: 49  Controls: 65 | SS + MS |  | 3dDMhead™ System  (3dMD, Atlanta, GA, USA) | Facial characteristics of SS and MS after operation |
| Tan (47) | 2013 | 3 | Retrospective cohort | 22 | UCS | FOA, ESC | Vectra 3D imaging  system (Canfield Imaging Systems) | Compare FOA and ESC |
| Toma (48) | 2010 | 3 | Retrospective cohort | 33 | SS | Melbourne method of Total Vault remodeling | 4-modular 12-camera + 5-modular 15-camera unit + 3D CT | Evaluate morphometric outcome of Melbourne procedure |
| Tu (49) | 2019 | 3 | Retrospective cohort | 28 | SS, MS, UCS | NA | 3dMD head System (3dMD, Atlanta, GA) | Calculate malformation at area of each cranial bone |
| Tu (50) | 2018 | 3 | Retrospective cohort | 17 | SS, MS, CS | NA | 3dMD head System (3dMD, Atlanta, GA) + 3D CT | to qualify cranial malformation from 3D photogrammetry |
| Van Veelen (51) | 2016 | 3 | Retrospective cohort | 95 | SS | ESC; total cranial remodeling | 3dMD head System (3dMD, Atlanta, GA) | Compare postoperative volume between ESC and total cranial vault |
| Varagur (52) | 2022 | 3 | Retrospective cohort | 35 | USC | Endoscopic VS open repair | 3dMDhead stereophotogrammetry  system (3dMD) | Asymmetry between endoscopic and open repair |
| Willebrand (53) | 2012 | 3 | Retrospective cohort | 28 | SS, MS, USC, lamboid | cranial vault  correction with or without FOA | Canfield VECTRA-  360-four-pod system | monitor feasibility of photogrammetry |
| Yen, D (54) | 2019 | 4 | Case-control | 25 | SS | modified pi surgical repair | 4-pod camera system (3dMD, Atlanta, GA) | Follow-up frontal bossing before and after frontal craniotomy |
| Zapatero, Z (55) | 2022 | 3 | Retrospective cohort | 17 | SS | Helmet, SAC | Polhemus FastScan Handheld Class I Laser Scanner | Effect of helmet in SS before springs |

^a^ patients with metopic ridge with and without trigonocephaly

ESC = Extended strip craniotomy, SAC = Spring assisted craniotomy, FOA = Fronto-orbital advancement, FOR = Fronto-orbital remodeling, SS = Sagittal synostosis, USC = Unicoronal synostosis, MS = Metopica synostosis, CS= coronal suture (both uni- and bilateral coronal synostosis), NSC = Non syndromic synostosis

**Supplementary** Table 2. Volume measurement

| **Author** | **Measurement** | **Landmarks for baseplane** | **Volume definition** |
| --- | --- | --- | --- |
| Abdel-Alim (2023) | Cranial Volume | Tragus (L+R) + nasion | Cranial volume: above plane (corrected based on CT-scan) |
| Abdel-Alim (2023) | Cranial Volume | Tragus (L+R) + nasion | Cranial volume: above plane |
| Al Shaqsi (2018) | Cranial Volume | Lower orbital (L+R) + Opisthocranion | Cranial volume: above plane |
| Applegren (2018) | Cranial Volume  Quadrants  ACV/PCV | Tragus (L+R) + sellion | Cranial volume: volume measurements from levels 2 to 8; level 0 (cranial base) to level 10 (vertex of head)  Anterior/Posterior Cranial Volume (ACV/PCV): Sum of Anterior or Posterior quadrants |
| de Jong (2017) | Cranial Volume  Anterior volume | Sella turcica^1^ + nasion | Cranial Volume: above plane  Anterior Volume: the volume in front of the plane crossing the center of the sella turcica perpendicular to both the sella turcica-nasion plane and the mid-sagittal plane |
| de Jong (2023) | Cranial volume | Sella turcica^1^ + nasion | Cranial Volume: above plane |
| Elawadly (2022) | APVR | Preaurales (L+R) + sellion | Anteroposterior volume ratio (APVR): anterior volume/posterior volume. |
| Elawadly (2023) | ASR | Preaurales (L+R) + sellion | Anterior Asymmetry Ratio (ASR):Q_max_/Q_min_ |
| Freudlsperger (2013) | Cranial volume Quadrants  ACV/PCV | Tragus (L+R) + subnasale | Cranial volume: above plane  Anterior/Posterior Cranial Volume (ACV/PCV): Sum of Anterior or Posterior quadrants |
| Kuehle (2022) | Cranial Volume Quadrants | Tragus (L+R) + nasion | Cranial volume: above plane |
| Le (2014) | Cranial Volume | Laterale canthus + tragus | Cranial volume: above plane |
| Linz (2014) | Cranial volume, Quadrants and ACAI/PCAI | Tragus (L+R) + nasion | Cranial volume: above plane  Anterior Cranial Asymmetry Index (ACAI): (Larger Q - Smaller Q)/Smaller Q x100  Posterior Cranial Asymmetry Index (PCAI): (Larger Q - Smaller Q)/Smaller Q x100 |
| McKay (2010) | Cranial Volume^2^ | Laterale canthus + tragus | Cranial volume: above plane |
| Medina (2023) | Cranial Volume  Anterior/posterior volume | Tragus (L+R) + nasion | Cranial volume: volume measurements from level 2 to 8; level 0 (skull base) to level 10 (total height)  Anterior/posterior vault volume: volume anterior and posterior to inter-tragi line |
| Mertens (2017) | Cranial Volume Quadrants | Tragus (L+R) + subnasale | Cranial volume: above plane |
| Seeberger (2016) | Cranial Volume and Quadrants | Tragus (L+R) + subnasale | Cranial volume: above plane |
| Sharma (2018) | Cranial Volume | Tragus (L+R) + nasion | Cranial volume: above plane |
| Van Veelen (2016) | Cranial Volume^2^ | Tragus + lateral canthus (L+R) | Cranial volume: above plane |
| Willebrand (2012) | Cranial Volume, Quadrants and ASR/PSA | Tragus (L+R) + subnasale | Cranial volume: above plane  Anterior Asymmetry Ratio (ASR):Q_max_/Q_min_  Posterior Asymmetry Ratio (PSR): Q_max_/Q_min_ |

^1^ Landmark Sella Turcica: Specific CCFP to sella turcica offset for orientating 3D photos in reference frame of CT-scan

^2^ Validated with CT

**Supplementary** Table 3. Width, height and length

| **Author** | **Landmarks baseplane** | **Measurement plane** | **Width** | **Length** | **Vertex height** |
| --- | --- | --- | --- | --- | --- |
| Abdel-Alim (2023)^1^ | Tragus (L+R) + nasion | At maximum length | Max width on plane | Max length on plane |  |
| Al-Shaqsi (2018)^1^ |  |  | tragus - tragus | glabella - opisthocranion | vertex - midline tragus |
| Al-Shaqsi (2021)^1^ |  |  | euryon - euryon | glabella - opisthocranion |  |
| Applegren (2018)^1^ | Tragus (L+R) + sellion | Level 3/10 | Max width on plane | Max length on plane |  |
| Borghi (2022)^1^ | Tragus (L+R) + nasion | - | Max width above plane | Max length above plane |  |
| De Jong (2023)^1^ | Cephalic length |  | Max width above plane | Max length above plane |  |
| Elawadly (2023)^1^ |  |  | euryon - euryon | glabella - opisthocranion |  |
| Fotouhi (2023)^1^ |  |  | euryon - euryon | glabella - opisthocranion |  |
| Klausing (2019)^1^ |  |  | euryon - euryon | glabella - opisthocranion |  |
| Kuehle (2022) | Tragus (L+R) + nasion | At ⅓ of the cranial height | Parallel to Tragus - Tragus |  |  |
| Le (2014) |  |  |  |  | vertex - nasion |
| Linz (2014)^1,2^ | Tragus (L+R) + nasion | At opisthocranion | Max width on plane | Max length on plane |  |
| Linz (2014)^2^ |  |  | tragus - tragus |  |  |
| Martini (2015)^1^ |  |  | euryon -euryon | glabella - opisthocranion |  |
| Medina (2023)^1^ | Tragus (L+R) + nasion | At level 3/10 | Max width on plane | Max length on plane |  |
| Mertens (2017)^1^ | Tragus (L+R) + subnasale | At level 5/11 | Max width on plane | Max length on plane |  |
| Nguyen (2023)^1^ |  |  | euryon - euryon | glabella - opisthocranion |  |
| Pickergill (2018)^1^ |  |  | euryon - euryon | glabella - opisthocranion | vertex - nasion |
| Ramamurthi (2021) |  |  |  |  | vertex - porion |
| Rodriquez (2017)^1^ | Tragus (L+R) + nasion |  | Max width above plane | Max length above plane |  |
| Rodriquez (2020)^1^ | Tragus (L+R) + nasion |  | Max width above plane | Max length above plane |  |
| Schulz (2021)^1^ | Tragus (L+R) + exocanthion | At maximum circumference | Max width on plane | Max length on plane |  |
| Seruya |  |  |  | glabella - opisthocranion |  |
| Sharma (2018)^1^ | Tragus (L+R) + nasion |  | Max width above plane | Max width above plane |  |
| Toma (2010)^1^ |  |  | euryon - euryon | glabella - opisthocranion | vertex - porion |
| Tu (2019)^1^ | Nasion + clinoid procosses dorsum sella^4^ | At maximum circumference | Max width on plane | Max length on plane |  |
| Willebrand (2012)^1,2^ | Tragus (L+R) + subnasale | At slice (of 11 levels) with maximum length | Max width on plane | Max length on plane |  |
| Willebrand (2012)^2^ | Tragus (L+R) + subnasale | Level 5/11 | Max width on plane | Max length on plane |  |
| Zapatero (2022)^1^ | Frankfort horizontal | At maximum circumference | Max width on plane | Max length on plane |  |
| Elawadly (2022)^3^ | Preaurales (L+R) + sellion | At glabella | frontal width at P1-P11/P2-10  posterior width at P5-P7/P4-P8 |  |  |

^1^ CI measured based on the width and length

^2^ Multiple measurements for width and length described

^3^ APWR1/APWR2 calculated with frontal and posterior width

^4^ approximated using a CT based program

**Supplementary** Table 4. Cranial asymmetry

| **Author** | **Measurement** | **Definition** | **Landmarks baseplane** | **Measurement Plane** | **Diagonals** |
| --- | --- | --- | --- | --- | --- |
| Applegren (2018) | CVAI | Cranial Vault Asymmetry index (CVAI): Percent difference between two diagonals | tragus (L+R) + sellion | Level 3/10 parallel to baseplane | 30° |
| Elawadly (2023) | CVAI | Cranial Vault Asymmetry index (CVAI): Percent difference between two diagonals |  | Axial plane through glabella and opisthocranion | 30° |
| Elawadly (2022) | APDR30/60 | anterioposterior diagonal ratio (APDR): ratio frontal length and posterior length in diagonals (30°/60°) | Preaurales (L+R) + sellion | At glabella parallel to baseplane | 30°/60° |
| Hallac (2023) | CVAI |  |  |  |  |
| Hallac (2023) | DD |  |  |  |  |
| Hallac (2023) | OCLR |  |  |  |  |
| Linz (2014) | DD | Diagonal difference (DD): differences between the two diagonals at the measurement plane | Tragus (L+R) + nasion | at opistocranion parallel to baseplane | 30° |
| Sakar (2022) | CVAI |  | Tragus (L+R) + sellion | Level 3/10 parallel to baseplane | 30° |
| Schulz | DI | 30° diagonals index (DI): (left + right 30° diagonal)/(2 × length) | Tragus (L+R) + exocanthions (L+R) | At maximum circumference parallel to baseplane | 30° |

**Supplementary** Table 5. Circumference

| **Author** | **Measurement** | **Landmarks baseplane** | **Measurement plane** |
| --- | --- | --- | --- |
| Abdel-Alim (2023) | Headcircumference | Tragus (L+R) + nasion | At maximum circumference parallel to baseplane |
| Abdel-Alim (2023) | Headcircumference | Tragus (L+R) + nasion | At maximum circumference parallel to baseplane |
| Applegren (2018) | Headcircumference | Tragus (L+R) + sellion | Level 3/10 parallel to baseplane |
| de Jong (2023) | Headcircumference |  | At crossing points of cranial width and length |
| Fotouhi (2023) | Headcircumference |  | Plane not described |
| Linz (2014)^1^ | Headcircumference | Tragus (L+R) + nasion | At opistocranion parallel to baseplane |
| Medina (2023) | Headcircumference | Tragus (L+R) + nasion | Level 3/10 parallel to baseplane |
| Mertens (2017) | Headcircumference | Tragus (L+R) + subnasale | Level 5/11 parallel to baseplane |
| Sakar (2022) | Headcircumference | Tragus (L+R) + sellion | Level 3/10 parallel to baseplane |
| Schulz (2021) | Headcircumference | Tragus (L+R) + exocanthions (L+R) | At maximum circumference parallel to baseplane |
| Seruya (2014) | Headcircumference |  | At glabella and opistocranion |
| Toma (2010) | Headcircumference |  | At glabella and opistocranion |
| Tu (2019) | Headcircumference | Nasion + clinoid processes dorsum sella* | At maximum circumference parallel to baseplane |
| Zapatero (2022) | Headcircumference | frankfort horizontal | At maximum circumference parallel to baseplane |
| Linz (2014)^1^ | Coronal circumference | ear - ear | Circumference over the top of the head from right ear landmark to left ear landmark, parallel to the coronal plane |
| Martini (2015) | Coronal circumference | ear - ear | Circumference over the top of the head from right ear landmark to left ear landmark, parallel to the coronal plane |
| Linz (2014)^1^ | Sagittal circumference | Tragus (L+R) + nasion | Circumference over the top of the sagittal plane starting at plane 0 at nasion |

* Use a CT framework to identify landmark

^1^ Multiple measurements for width and length described

**Supplementary** Table 6. Cranial shape

| **Author** | **Measurement** | **Definition** |
| --- | --- | --- |
| Chou (2017) | Heatmaps | Show changes overtime pre-operative and post-operative. |
| de Jong (2017) | Heatmaps | Heatmaps were generated to show the absolute and normalized change between two sequential age groups. |
| de Jong (2023) | Heatmaps | Color-coded growth maps visualizing the difference between mean head shapes in two sequential age groups or between treatments groups |
| Harrison (2023) | Heatmap + Curvature analysis | Heatmaps were generated to compare pre-operative and post-operative images.  Curvature analysis of the forehead and temple areas |
| Nguyen (2023) | Heatmap | Difference in surface distance between pre- and post-band images. Additionally, root mean square calculated for eight separate regions (absolute average distance) |
| Porras (2019) | Heatmap:  malformation + curvature discrepancies | Malformation: local distances between the patient's head and its matched normal head  Curvature discrepancies: local curvature differences  Quantification with CT |
| Ramamurthi (2021) | Heatmaps | Distance in surface between patients and control |
| Rizvi (2023) | Heatmaps | Distance between surface on pre- and post-operative images and their corresponding age-matched control |
| Rodriguez-Florez (2020) | Heatmaps | Using non-parametric SSM framework (Deformetica): computes the mean shape and the deformation vectors. Comparing pre-op, post-op, follow-up and removal population |
| Rodriguez-Florez (2017) | Heatmaps | Using non-parametric SSM framework (Deformetica): computes the mean shape and the deformation vectors. Comparing pre-op, post-op and control population |
| Sharma (2018) | Heatmaps | Distances between pre-op and post-op images |
| Schulz (2021) | Heatmaps | Volume gain/loss pre- versus post-operative and pre-operative versus control |
| Tu (2019) | Heatmaps | Using PCA and Euclidean distance to measurement each point closest to the normal shape |
| Tu (2018) | Heatmaps | Using PCA and Euclidean distance to measurement each point closest to the normal shape |
| Cho (2018) | Curvature analysis | Curvature analysis and K-means cluster analysis to classify into groups. In addition, focus on 3 regions of interest (metopic strip, both temporal regions). |
| Borghi (2022) | Principal component analysis | PCA captures the shape variance around the template by extracting the deformations required to turn the template shape back to each individual shape. Each subject can be described by the mean shape and specific shape deformation vectors (variation modes). Controls used (n=6) |
| Heutinck (2021) | Principal component analysis | PCA describes the shape variance around the template by extracting the deformations required to turn the template shape back to each individual shape (shape vectors). Controls used (n=65) |
| Meulstee (2017) | Principal component analysis | PCA for diagnosis  Using PCA to find areas of most variation to distinguish between craniosynostosis |
| Schaufelberg (2022) | Classification pipeline | Statistical shape modeling and PCA to create a classification pipeline for craniosynostosis types |
| Kronig (2022) | Sinusoid curves | Sinusoid curves of cranial shape which show patterns for craniosynostosis |
| Kronig (2023) | Sinusoid curves | Sinusoid curves of cranial shape which show patterns for craniosynostosis |
| Elawadly (2022) | Anteroposterior area ratio (APAR) | The differential growth in scalp surface area between front and back half of neurocranium. Superior to base plane (pre-aurales + sellion). |

**Supplementary** Table 7. Forehead

| **Author** | **Measurement** | **Goal** | **Definition** |
| --- | --- | --- | --- |
| Rodriquez-Florez (2017) | Forehead volume | Forehead prominence | Volume between baseplane (Crus helix (L+R) + nasion) and posterior platform at 120 degrees |
| Rodriquez-Florez (2020) | Forehead volume | Forehead prominence | Idem to Rodriquez-Florez (2017) |
| Yen (2019) | Prenasion volume | Forehead prominence | Volume nasion to perpendicular› vertex |
| Le (2014) | Forehead inclination | Forehead prominence | Angle between a transverse line through the nasion that was parallel to Frankfort horizontal and a line running between glabella and nasion |
| Yen (2019) | Forehead inclination | Forehead prominence | Angle between nasion parallel to frankfort horizontal and line between glabella and nasion |
| Kuehle (2022) | Nasofrontal angle | Forehead prominence | Angle between glabella-Nasion-Prenasale |
| Schulz (2021) | Nasofrontal angle | Forehead prominence | Angle between back of the nose to glabella |
| Badiee et al (2022) | Frontal width | Width | Distance between frontotemporal points |
| Klausing (2019) | Frontal width | Width | Distance between frontotemporal points |
| Pfaff (2023) | Frontal width | Width | Distance between frontotemporal points |
| Seruya (2014) | Frontal width | Width | Distance between frontotemporal points |
| Kuehle (2022) | Interfrontoparietal-  interparietal ratio | Width | Idem to Rodriguez-Florez (2017) |
| Rodriguez-Florez (2017) | Interfrontoparietal-  interparietal ratio | Width | Idem to Rodriguez-Florez (2017) |
| Rodriguez-Florez (2017) | Interfrontoparietal-  interparietal ratio | Width | Ratio between Interparietal distance (BC: width at measurement plane) and interfrontoparietal distance (DE: halfway between glabella and Interparietal distance)  Measurement plane: 1/3 of head height at baseplane (L + R crus helix + Nasion) |
| Elawadly (2022) | FA30 | Forehead Wedging | 30 degrees diagonal frontal angle between vector P.11-P.12 and P12.P1 at measurement plane. Measurement plane: at glabella parallel to baseplane (preaurales (L+R) + sellion) |
| Leclair (2023) | Anterior Arc Angle | Forehead Wedging | 30 degree lines to intersection with forehead left and right of skull centroid. Angle between anterior most point and intersections. Measurement plane: plane of maximum circumference above glabella |
| Rodriguez-Florez (2017) | Frontal angle | Forehead Wedging | Angle between DAE  A: glabella.  BC: interparietal distance: width at measurement plane  D/E: halfway AB/AC  Measurement plane: 1/3 of head height at baseplane (L + R crus helix + Nasion) |
| Klausing (2019) | Frontal angle | Forehead Wedging | Idem to Martini (2015) |
| Martini (2015) | Frontal angle | Forehead Wedging | Angle between right frontotemporal point, glabella and left frontotemporal point  Frontotemporal point: perpendicular to L+R exocanthion at measurement plane  Measurement plane: parallel to the Frankfort horizontal plane at the level of the glabella. |
| Martini (2017) | Frontal angle | Forehead Wedging | Idem to Martini (2015) |
| Kuehle (2022) | Frontal angle | Forehead Wedging | Idem to Rodriguez-Florez (2017) |
| Badiee et al. (2022) | Glabellar angle | Forehead Wedging | Angle between Zygomaticofrontal left (ZFl) -Glabella- Zygomaticofrontal right (ZFr)  ZFr/ZFl: perpendicular to right/left ZF suture at measurement plane  Measurement plane: parallel to the Frankfort horizontal plane at the level of the glabella. |
| Klausing (2019) | Frontoparietal angle L/R | Temporal hollowing | Idem to Martini (2015) |
| Martini (2015) | Frontoparietal angle L/R | Temporal hollowing | Angle between L/R parietal point, L/R frontotemporal point and glabella  Parietal point: perpendicular to L/R preaurales at measurement plane  Frontotemporal point: perpendicular to L+R exocanthion at measurement plane  Measurement plane: parallel to the Frankfort horizontal plane at the level of the glabella. |
| Martini (2017) | Frontoparietal angle L/R | Temporal hollowing | Idem to Martini (2015) |
| Sakar (2022) | Area under sinusoidal curve | Frontal shape | Distance between midpoint (halfway anterior-posterior) to the outerline of left (0°) to right (180°) temporal region |
| Pressler (2021) | Axial contours of the forehead | Frontal shape | Distances at 15° increments between origin and axial contour at level of origin  Origin: intersection of points 20 mm above glabella, and 20 mm above L+R helical root |
| Varagur (2022) | Forehead symmetry | Frontal shape | The right and left anterior portions of the upper skull were isolated and split at the midsagittal plane. The image was cropped inferior to the left inferior medial brow line and posterior to the left tragus. A mirror image of the right side was generated and overlaid on the left side. The root mean square distance was calculated between the non-fused side and the fused side. |
| Linden (2019) | Vector analysis technique | Frontal shape | Distances between origin to surface of the face at 9 soft tissue landmarks (spaced every 10 mm) at level of origin.  Origin: positioned in a horizontal plane 60 mm behind the surface of the face an 20 mm above the glabella |

**Supplementary** Table 8. Face

| **Author** | **Year** | **Type** | **Measurement** | **Definition** |
| --- | --- | --- | --- | --- |
| Gabrick | 2020 | Asymmetry analysis (mirroring) | Procrustes analysis | After the image is registered on a Euclidean plane, a mirror image was superimposed, and the root mean square (RMS) distance between the 2 images are calculated among multiple points on the face.  Analysis of: total asymmetry, facial vertical thirds, areas of known deformities (brow, orbit, nose) |
| Owall | 2016 | Asymmetry analysis (mirroring) | Asymmetry measurement | Measuring distances between points on the surface and corresponding points on the mirrored version of the same surface (Vector A). Heatmaps of asymmetry in transverse, sagittal and vertical direction.  Analysis of: total face and 6 subregions (forehead, mouth, eyes, nose, cheek, and chin) |
| Owall | 2019 | Asymmetry analysis (mirroring) | idem Owall 2016 | idem Owall (2016) |
| Svalina | 2022 | Asymmetry analysis (mirroring) | Facial symmetry parameters | For measuring symmetry parameters, the facial surface was mirrored across the XY plane (mid-sagittal plane). The face was divided into five different regions.  Analysis of: whole face, forehead, eyes-nose, nose-lips, chin, eyes-lips |
| Tan | 2013 | Angle | Facial deviation | Angle between midline (perpendicular to endocanthia line at origin) and origin to gnation. Origin: halfway between endocanthia |
| Varagur | 2022 | Angle | Facial midline deviation | Idem to Oh (2008) |
| Oh | 2008 | angle | Facial midline deviation | Angle between midline (perpendicular to the endocanthia through the sellion) and sellion to gnation |
| Tan | 2013 | Angle | Nasal tip deviation | Angle between midline (perpendicular to endocanthia line at origin) and origin to prenasale. Origin: halfway between endocanthia |
| Oh | 2008 | Angle | Nasal tip deviation | Angle between midline (perpendicular to the endocanthia through the sellion) and sellion to prenasale |
| Varagur | 2022 | Angle | Nasal tip deviation | Idem to Oh (2008) |
| Schulz | 2021 | Ratio | Exocanthion index | Midpoint to left + right exocanthion distance/(2x anterior part of the length) |

**Supplementary** Table 9. Anthropometric measurements

| **Author** | **Year** | **Measurement** |
| --- | --- | --- |
| Varagur | 2022 | Endocanthion - sellion |
| Svalina | 2022 | Endocanthion - midline |
| Svalina | 2022 | Endocanthion - endocanthion |
| Badiee et al. | 2022 | Endocanthion - endocanthion |
| Kuehle | 2022 | Endocanthion - endocanthion |
| Kuehle | 2022 | Exocanthion - exocanthion |
| Svalina | 2022 | Exocanthion - exocanthion |
| Oh | 2008 | Exocanthion - sellion |
| Tan | 2013 | Exocanthion - sellion |
| Svalina | 2022 | Exocanthion - sellion |
| Svalina | 2022 | Christa Philtri - sellion |
| Oh | 2008 | Tragus - gnathion |
| Tan | 2013 | Tragus - gnathion |
| Varagur | 2022 | Tragus - gnathion |
| Oh | 2008 | Tragus - sellion |
| Tan | 2013 | Tragus - subnasale |
| Varagur | 2022 | Tragus - subnasale |
| Svalina | 2022 | Tragus - midline |
| Svalina | 2022 | Pogonion |
| Svalina | 2022 | Labiale inferius |
| Svalina | 2022 | Labiale superus |
